# Association between childhood adversity and a diagnosis of personality disorder in young adulthood: a cohort study of 107,287 individuals in Stockholm County

**DOI:** 10.1007/s10654-017-0264-9

**Published:** 2017-05-30

**Authors:** Emma Björkenstam, Lisa Ekselius, Bo Burström, Kyriaki Kosidou, Charlotte Björkenstam

**Affiliations:** 1 Division of Social Medicine, Department of Public Health Sciences, Karolinska Institutet, Stockholm, Sweden; 2 Department of Community Health Sciences, California Center for Population Research, Fielding School of Public Health, University of California, Los Angeles, Los Angeles, CA USA; 3 Department of Neuroscience, Psychiatry, Uppsala University, Uppsala, Sweden; 4 Division Public Health Epidemiology, Department of Public Health Sciences, Karolinska Institutet, Stockholm, Sweden; 5 Center for Epidemiology and Community Medicine, Stockholm County Council, Stockholm, Sweden; 6 Department of Epidemiology, Fielding School of Public Health, University of California, Los Angeles, Los Angeles, CA USA; 7 Department of Clinical Neuroscience, Insurance Medicine, Karolinska Institutet, Stockholm, Sweden; 8 Department of Sociology, Stockholm University, Stockholm, Sweden

**Keywords:** Childhood adversity, Personality disorder, Epidemiology, Cohort, Sweden

## Abstract

Childhood adversity (CA) may increase the risk for later developing of personality disorder (PD). However, less is known about the association between cumulative CA and PD, and the role of childhood psychopathology and school performance. The current study examined the relationship between a range of CAs and a diagnosis of PD in young adulthood, and the roles of childhood psychopathology and school performance in this relationship. All individuals born in Stockholm County 1987–1991 (n = 107,287) constituted our cohort. Seven CAs were measured between birth and age 14: familial death, parental criminality, parental substance abuse and psychiatric morbidity, parental separation and/or single-parent household, household public assistance and residential instability. Individuals were followed from their 18th birthday until they were diagnosed with PD or until end of follow-up (December 31st 2011). Adjusted estimates of risk of PD were calculated as hazard ratios (HR) with 95% confidence intervals (CI). Associations were observed between cumulative CA and PD. During the follow-up 770 individuals (0.7%) were diagnosed with PD. Individuals exposed to 3+ CAs had the highest risks of being diagnosed with PD (HR 3.0, 95% CI 2.4–3.7). Childhood psychopathology and low school grades further increased the risk of PD among individuals exposed to CA. Cumulative CA is strongly associated with a diagnosis of PD in young adulthood. Our findings indicate that special attention should be given in schools and health services to children exposed to adversities to prevent decline in school performance, and to detect vulnerable individuals that may be on negative life-course trajectories.

**Electronic supplementary material:**

The online version of this article (doi:10.1007/s10654-017-0264-9) contains supplementary material, which is available to authorized users.

## Introduction

Personality disorders (PD) constitute a global health problem, and it is estimated to affect more than 5% of adults globally [1, 2]. PDs have a number of common features as well as diametrically different characteristics, and are organized into 10 categories in DSM-V [3] and 8 in ICD-10 [4]. Based on clinical utility and symptoms, they are grouped into the following clusters in DSM-V: cluster A; characterized by odd and eccentric behaviors, cluster B; by dramatic and erratic behaviors and cluster C; by anxious and fearful behaviors. Additionally, a substantial number of individuals have disorders of personality functioning that do not fulfil criteria for a defined PD, categorized as “other PD”. PDs typically have their onset in adolescence or early adulthood, and are generally persistent over time [5]. It is well established that individuals with a PD have higher morbidity and mortality rates compared with the general population [1, 2, 6, 7].

A major goal of the epidemiological investigation of PDs is to identify modifiable risk factors; early interventions against these factors may then reduce the incidence and severity of the disorder and its consequences. Childhood adversities (CA), especially abuse and neglect, have been pointed out as risk factors for PD [5, 8–12], and particularly for borderline PD (BPD) and antisocial PD [13, 14]. Other CAs that have been linked to PD include substance abuse in the home, a mentally ill household member or incarcerated parents, low family socioeconomic status and parental loss [15–17]. Childhood emotional and behavior problems have also been shown to be associated with greater risk for PD, as well as disruptive disorders during adolescence [18, 19].

With a few exceptions [9, 15], studies examining the effect of CA, including household substance abuse and mental disorder, parental divorce, and criminal household member, on PD have generally been based on clinical samples [20, 21], and longitudinal follow-up studies have been warranted [15]. Furthermore, most previous studies have used retrospectively self-reported adversities and are, thus, limited by recall bias [15, 20, 21].

Studies on CAs as risk factors for psychiatric disorders have shown that these indicators tend to occur in clusters rather than as single events, and that their cumulative impact on psychiatric disorders is substantial [22–24]. Whether this dose–response relationship is true for PD remain inadequately understood. Although the co-occurrence of multiple CAs is common [25], to the best of our knowledge, no prior study has examined if certain clusters of CAs can be identified as more closely related to PD.

Exposure to CA may negatively affect school performance and later educational attainment [26–28], and increases the risk of childhood psychopathology [29]. Low school performance and childhood psychopathology are both independent risk factors for PD [5, 30–32] and could partly explain the association between CA and PD. In addition, low cognitive ability, that is strongly related to school performance, and childhood psychopathology is an indication of vulnerability that might increase individuals’ susceptibility to detrimental environmental influences including CA. It could therefore be hypothesized that school performance and the development of childhood psychopathology might modify the CA-PD relationship but this has not previously been explored.

We used a large population-based cohort to examine the relationship between a prospectively recorded indicators of CA and PD. The seven adversities were familial death, parental criminality, parental substance abuse, parental separation and/or single-parent household, parental psychiatric morbidity, household public assistance, and residential instability. All these CAs have been demonstrated to have significant adverse health or social implications [15, 16, 22, 33–36].

We capitalized on Sweden’s extensive and high quality registers and conducted a population-based cohort study including approximately 107,000 individuals born between 1987 and 1991 in Stockholm County, Sweden. We aimed to explore the following research questions:

1. Does cumulative CA increase the risk of being diagnosed with PD in young adulthood?
2. To what extent does comorbidity with other psychiatric disorders in childhood or adolescence explain the association between CA and diagnosed PD?
3. Is the effect of CA on diagnosed PD modified or mediated by school performance?
4. Which clusters of CA are more closely related to PD?

## Methods

### Study population

The study population was defined as all individuals born in Stockholm County, Sweden between 1987 and 1991 (n = 116,087), obtained from the Medical Birth Register [37]. We excluded children who were adopted (n = 50), died before their 18th birthday (n = 947), and who emigrated (n = 6665). Furthermore, we excluded children who received disability pension (DP) at the age of 18 (n = 1138). Here we followed the example of a Norwegian register study on DP [38] to exclude the youngest disability pensioners, mainly persons with severe learning disabilities or multi-handicaps.

The unique personal identity number assigned to Swedish residents was used to link this cohort to multiple population-based registers as described below.

The Causes of Death Register comprises information on all deaths of Swedish residents. The National Patient Register (NPR) includes all individuals admitted to psychiatric or general hospitals, with complete coverage for inpatient care since 1987 and outpatient care since 2001 [39]. In addition, an administrative health care database (VAL) was used, containing individual data on utilization of publicly funded inpatient and outpatient health care in Stockholm County since 1997. The Total Enumeration Income Survey contains data on the income and governmental benefits provided to Swedish residents. The Total Population Register includes information on age, sex, place of residence, and other relevant demographic characteristics. The Longitudinal Integration Database for Health Insurance and Labor Market Studies register integrates existing data from the labor market, educational and social sectors. The Register of Court Convictions contains information on all court convictions in Sweden for persons 15 years of age or older. The National School Register (NSR) holds information on individual school performance (grade points by subject) for all students from the final ninth year in primary schools since 1988. Non-public schools, which comprise a few percent of all Swedish schools, have been included since 1993. The quality of the data in the NSR is high [40], and missing data is mostly due to lack of reporting from certain private schools. Families were linked together through the Multi-Generation register, which contains all known relationships between children and parents (born in 1932 or later) since 1961.

### Indicators of CA

The seven adversities are defined in Table 1. If more than one of the same CA occurred, the date of the first event was considered.

**Table 1:** Definitions and classification of childhood adversities

Indicator of childhood adversity	Definition	ICD classification	Data source
Familial death	Parental or sibling death	N/A	Causes of Death Register
Parental criminality	Parent sentenced to prison, probation, or forensic psychiatric care	N/A	Register of Court Convictions
Parental substance abuse	Parental hospitalization for alcohol and/or narcotic-related substance abuse, or parental alcohol or narcotic-related drug conviction	ICD-9: 291–292, 303–3050, 3570, 4255, 5353, 5710, 5711–5713, 6483, 6555, 9650, 9696–9697	National Patient Register
		ICD-10: E244, F10–F16, F18–F19, G312, G621, G721, I426, K292, K70, K852, K86, O354–355, P044, T40, T436, T51, Z502–503, Z714, Z721–Z722	Register of Court Convictions
Parental separation and/or single-parent household	Either having parents separated or living in a single-parent household, or both	N/A	Longitudinal Integration Database for Health Insurance and Labor Market Studies
Parental psychiatric morbidity	Parental hospitalization for mental disorder (excluding substance-abuse related disorders)	ICD-9: 290–319	National Patient Register
		ICD-10: F00–F99	
Household receiving public assistance	Public assistance during at least one year, where more than 50% of the yearly income constituted public assistance	N/A	Total Enumeration Income Survey
Residential instability	Two or more changes in place of residence	N/A	Total Population Register

### Personality disorder

PD was defined as having received at least one primary diagnosis of PD (ICD-10: F60.0-F60.9, according to the 10th International Classification of Disease, ICD-10) 2005–2011 in either in- or outpatient care, reported in the NPR or the VAL database. Due to small numbers, we collapsed the different types of PD and did not study them separately.

### Confounders, mediators and moderators

Our analyses adjusted for birth year, sex and whether or not the mother was born in Sweden. We also adjusted for parental education and disposable income, measured when the child was 15 years old (i.e. between 2002 and 2006). Parental educational level was classified into three categories: (1) Nine years or less, (2) 10–12 years, and (3) 13 years or more. Disposable income was assessed using the individualized weighted average family income.

Childhood and adolescent psychopathology (PD excluded) was defined as any inpatient and/or outpatient treatment with a psychiatric diagnosis before age 18 years (Chapter F excluding F60 in ICD-10), recorded in the NPR and/or VAL database. We also identified individuals receiving a psychiatric disorder after 18 years (PD diagnosis excluded).

School performance was based on the grade point average (GPA) from the final (ninth) year of compulsory school. The GPA was based on the student’s 16 best subjects. The child earned 10–20 points per passed subject, yielding a total maximum grade point of 320 points. Thus, the GPA spanned from 0 to 20 (as failed subjects did not yield any points).

### Statistical analysis

We performed multivariate analyses, using Cox hazards models, and calculated hazard ratios (HR) with 95% confidence intervals (CI). When modeling a Cox proportional hazard model a key assumption is proportional hazards. Among the various options for testing proportionality, we used the *estat phtest* command in Stata, and also the *tvc* and the *texp* options in the *stcox* command, followed by an LR test [41]. These tests did not indicate violation of the proportionality assumption (i.e. *p* values >0.05).

Four regression models were examined: Model I adjusted for sex and birth year. Model II was additionally adjusted for mother’s country of birth, parental income and education. Model III we added childhood and adolescent psychopathology. Finally Model IV was further adjusted for GPA. To assess cumulative effects, the total number of CAs were summed up and grouped into: 0, 1, 2, and 3 or more indicators.

For evaluating biological interaction [42] between CA and childhood and adolescent psychopathology, individuals were categorized as follows: Cumulative CA was here reduced to three groups (0, 1, and 2+), and we further dichotomized these three groups into having had a psychiatric in- or outpatient contact before age 18 or not. Hence, in total we had six mutually exclusive groups. In order to examine the modifying role of school performance, we used the GPA quartiles to create the following groups: “lowest grade group” (incomplete grades and the lowest quartile), “middle grade group” (second/third quartiles), and “highest grade group” (fourth quartile). Those with missing information on GPA were excluded from this analysis.

Among the different procedures to examine mediation, we employed the bootstrap method. This method, used to estimate the indirect effects in simple mediation models [43], is considered a powerful approach for estimating mediation and indirect effects. With the SAS PROCESS macro [44], we used a non-parametric bootstrapping method with 5000 resamples to derive the 95% confidence interval to test for the mediation effect [45]. We first regressed the mediator (GPA) on the independent variable (IV), i.e., cumulative CA using Ordinary Least Squares Regression (OLS). Second the dependent variable (DV), i.e., PD, was regressed on the IV, using logistic regression. Third, the DV was regressed on both the IV and the mediator, using logistic regression. Mediation was considered to occur if the relationship between the IV and DV could be partially or totally accounted for by the hypothesized mediator and if significant indirect effects were demonstrated [46].

In order to identify clusters of CA, we conducted Latent Class Analysis (LCA), using Latent Gold 4.5 (Statistical Innovations Inc., Belmont, MA). The goal of LCA is to identify the smallest number of latent classes that adequately describes the association among the observed indicators [47]. There are several strategies available to determine the number of classes [48, 49]. Since the sample size was large (N > 100,000), which makes *p* value-based significance testing less informative [50], we used the Bayesian information criterion (BIC) as our primary tool for determining the optimal number of classes. In addition, we also examined the reduction in L^2^, and the classification error in order to determine the best-fitting model [47, 48], and chose a 6-class model (Supplementary Table 1). The next step was to determine whether there were significant differences in HRs across the LCA classes. The modal assignment rule was used to assign cases to classes [47]. In these analyses, Models I-IV were repeated.

Statistical analyses were conducted using SAS v.9.4 and Stata v. 13.

## Results

Table 2 presents the characteristics of the cohort and the prevalence of CA and diagnosed PD. Fifty-one percent had experienced at least one CA, 27% had one CA, 14% two CAs, and 10% three or more. “*Parental separation and/or single household”* was the most common CA, experienced by 44%, followed by “*Household receiving public assistance”* (19%).

**Table 2:** Distribution of background variables, childhood adversity, and relation to PD diagnosis

	Total	Any PD diagnosis
N	107,287 (100)	770 (100)
*Child characteristics, n (%)*		
School performance		
Grade point average (GPA, SD)	14.1 (2.9)	13.1 (2.7)
Missing	3417 (3)	42 (5)
Childhood/adolescent psychopathology [psychiatric disorder before age 18 (excluding PD diagnoses)]	7289 (7)	250 (32)
Psychiatric disorder after age 18 (excluding PD diagnoses)	8827 (8)	472 (61)
*Parental characteristics*		
Parental educational level		
9 years of education	6322 (6)	50 (6)
10–12 years of education	46,042 (43)	371 (48)
>12 years of education	54,792 (51)	340 (44)
Missing	131 (0)	9 (1)
Average annual family income in Swedish Krona (SD)	438,050 (541,843)	368,234 (399,342)
Mother born outside of Sweden	20,481 (19)	135 (18)
*Indicators of childhood adversity*		
Familial death	3456 (3)	34 (4)
Parental criminality	4572 (4)	64 (8)
Parental substance abuse	9173 (9)	118 (15)
Parental separation and/or single-parent household	47,467 (44)	468 (61)
Parental psychiatric morbidity	5437 (5)	78 (10)
Household receiving public assistance	20,899 (19)	259 (34)
Residential instability	4551 (4)	63 (8)
*Total number of childhood adversities*		
0	52,698 (49)	246 (32)
1	29,209 (27)	223 (29)
2	14,935 (14)	154 (20)
3+	10,445 (10)	147 (19)

Absolute numbers and column percent

*SD* standard deviation

In the entire cohort, 0.7% received a PD diagnosis during the follow-up period. Diagnosed PD was much more common in women [of the 770 PD cases, 78% were females (data not shown)]. Among PD patients, Cluster B diagnoses were most common (62%) (data not shown).

One third of those who received a PD diagnosis had been treated in psychiatric care with a differential diagnosis before their 18th birthday. Approximately 25% had received a diagnosis for anxiety disorder, and another 25% for substance abuse disorders (data not shown). Nearly two thirds of the 770 PD patients received additional psychiatric diagnoses during the follow-up period. The most common comorbid diagnoses were anxiety disorders (26%), and substance abuse disorders (24%) (data not shown). CA was highly prevalent among those who received a PD diagnosis. Approximately 60% of the PD patients experienced a parental separation or grew up in a single-parent household, and around 15% grew up with substance abusing parents.

The results in Table 3 show an increased risk for PD in individuals exposed to CA, with a steep rate increase as the number of adversities increased. The crude HRs (adjusted only for birth year and sex) displayed elevated risk for PD for all CAs, with highest HRs for parental psychiatric morbidity and household public assistance (Table 3, Model I^a^). A gradual increase in PD risk was noted as the number of CAs increased. Individuals exposed to three or more adversities had a threefold risk of PD (HR 3.0, 95% CI 2.4–3.7), in the crude model. Adjustments in the second model had little effect on the HRs (Model II^b^). In the third model, the gradient still remained, although with lower HRs. Finally in the fully adjusted model (Model IV^d^), all HRs decreased further, suggesting that a large part of the association was explained by school performance.

**Table 3:** Associations between childhood adversity (CA) and diagnosed personality disorders (PD)

	Rates of PD (No/100,000 person years)	Model I^a^	Model II^b^	Model III^c^	Model IV^d^
Familial death	198.3 (137.3–277.1)	1.4 (1.0–1.9)	1.1 (0.8–1.6)	1.0 (0.7–1.5)	0.9 (0.6–1.4)
Parental criminality	278.7 (214.7–355.9)	2.0 (1.5–2.6)	1.8 (1.3–2.3)	1.5 (1.2–2.0)	1.4 (1.1–1.9)
Parental substance abuse	262.9 (217.6–314.8)	1.9 (1.6–2.3)	1.7 (1.4–2.1)	1.4 (1.2–1.8)	1.3 (1.1–1.6)
Parental separation and/or single-parent household	203.7 (185.6–223.0)	2.0 (1.7–2.3)	1.8 (1.5–2.1)	1.6 (1.3–1.8)	1.4 (1.2–1.7)
Parental psychiatric morbidity	295.1 (233.3–368.3)	2.1 (1.7–2.7)	1.9 (1.5–2.5)	1.6 (1.2–2.0)	1.4 (1.1–1.9)
Household receiving public assistance	250.0 (220.5–282.4)	2.1 (1.8–2.4)	1.9 (1.7–2.3)	1.6 (1.4–1.9)	1.4 (1.2–1.7)
Residential instability	285.2 (219.1–364.9)	2.0 (1.5–2.6)	1.8 (1.4–2.3)	1.5 (1.1–1.9)	1.3 (0.9–1.7)
*Total number of CAs*					
0	95.2 (83.6–107.8)	1 (REF)	1 (REF)	1 (REF)	1 (REF)
1	158.5 (138.4–180.7)	1.6 (1.4–2.0)	1.6 (1.3–1.9)	1.5 (1.2–1.8)	1.4 (1.1–1.7)
2	211.1 (179.1–247.2)	2.2 (1.8–2.7)	2.1 (1.7–2.6)	1.8 (1.4–2.2)	1.5 (1.2–1.9)
3+	285.3 (241.0–335.3)	3.0 (2.4–3.7)	2.7 (2.2–3.4)	2.1 (1.7–2.6)	1.7 (1.3–2.2)

Hazard ratios (HR) with 95% confidence intervals (CI)

^a^Adjusted for birth year and sex

^b^Model I with additional adjustments for mother’s country of birth, parental income and education

^c^Model II with additional adjustments for childhood/adolescent psychopathology [i.e. psychiatric diagnosis before age 18 years (PD excluded)]

^d^Model III with additional adjustments for grade point average

Childhood and adolescent psychopathology tended to modify the association between CA and diagnosed PD (Table 4). Highest risk for being diagnosed with PD was observed in those with a history of childhood and adolescent psychopathology, who were exposed to multiple CAs. HRs remained significantly elevated after controlling for school performance.

**Table 4:** Association between childhood adversity (CA), childhood/adolescent psychopathology, school performance, and diagnosed personality disorder (PD)

	n cases (any PD)	Rates of PD (No/100,000 person years)	Model I^a^	Model II^b^
*CA and childhood/adolescent psychopathology*				
No CA, no childhood/adolescent psychopathology	187	75.6 (65.1–87.2)	1 (REF)	1 (REF)
1 CA, no childhood adolescent psychopathology	153	116.1 (98.4–136.0)	1.5 (1.2–1.9)	1.4 (1.1–1.8)
2+ CAs, no childhood adolescent psychopathology	180	161.1 (138.4–186.5)	2.1 (1.7–2.6)	1.7 (1.3–2.1)
No CA, childhood adolescent psychopathology	59	532.4 (405.3–686.7)	6.9 (5.1–9.2)	6.7 (5.0–9.0)
1 CA, childhood adolescent psychopathology	70	783.8 (611.0–990.3)	10.1 (7.7–13.3)	8.2 (6.1–11.1)
2+ CAs, childhood adolescent psychopathology	121	949.0 (787.4–1133.9)	12.5 (9.9–15.7)	9.2 (7.1–12.0)

	n cases (any PD)	Rates of PD (No/100,000 person years)	Model I^a^	Model II^c^
*CA and school performance (n* *=* *103,380)*				
No CA, highest grade group	33	46.6 (32.1–65.5)	1 (REF)	1 (REF)
1 CA, highest grade group	25	93.1 (60.2–137.4)	1.9 (1.1–3.2)	1.8 (1.1–3.1)
2+ CAs, highest grade group	23	184.5 (117.0–276.9)	3.7 (2.2–6.3)	3.5 (2.0–5.9)
No CA, middle grade group	115	95.0 (78.5–114.1)	2.5 (1.7–3.6)	2.5 (1.7–3.6)
1 CA, middle grade group	81	132.2 (105.0–164.3)	3.3 (2.2–4.9)	3.2 (2.1–4.8)
2+ CAs, middle grade group	72	171.3 (134.1–215.8)	4.1 (2.7–6.1)	3.8 (2.4–5.7)
No CA, lowest grade group	91	149.0 (120.0–183.0)	4.6 (3.1–6.8)	4.2 (2.8–6.3)
1 CA, lowest grade group	111	229.4 (188.7–276.2)	6.7 (4.5–9.8)	5.5 (3.7–8.3)
2+ CAs, lowest grade group	177	279.8 (240.1–324.2)	7.7 (5.3–11.2)	6.0 (4.0–8.9)

Hazard ratios (HR) with 95% confidence intervals (CI)

^a^Adjusted for birth year and sex

^b^Model I with additional adjustments for mother’s country of birth, parental income and education, and school performance

^c^Model II with additional adjustments for childhood/adolescent psychopathology [i.e. psychiatric diagnosis before age 18 years (PD excluded)]

Similarly, exposure to both CA and poor school performance further increased the risk of diagnosed PD (Table 4). Individuals exposed to 2+ CAs and poor school performance had a sixfold risk for diagnosed PD (HR: 6.0, 95% CI 4.0–8.9).

In the mediation analyses (see supplementary Figure 1), cumulative CA (IV) predicted GPA (mediator) (β = −0.7, *p* < .0001). In the second step, cumulative CA (IV) predicted PD (DV) (β = 0.30, *p* < .0001). In the third step, which included all three variables (IV, DV and mediator), the relationship of cumulative CA (IV) with PD (DV) decreased, though remained significant. The mediation analysis based on the bootstrap method estimated the indirect effect of cumulative CA on PD through the mediator (i.e. GPA) to be 0.06 (95% CI 0.04–0.08), indicating the presence of small but significant indirect effect of GPA.

Supplementary Figure 2 depicts the identified classes of CAs along with the conditional probabilities for each of the exposure variables. The classes were labeled according to the levels of the conditional probabilities. Around 54% (n = 58,750) of the cohort were assigned to class 1. This class together with class 2 is mainly characterized by individuals with no CA, and therefore, the conditional probabilities for the exposure variables are more or less zero for all adversities. Class 3 is characterized by people who were mainly exposed to parental separation/single-parent household and public assistance (11%, n = 11,855). Class 4 represents individuals mainly exposed to parental substance abuse, separation/single-parent household and public assistance (5%, n = 5437). Class 5 is characterized by individuals with a history of parental substance abuse, separation/single-parent household and psychiatric morbidity (0.8%, n = 832). Finally, class 6 represents individuals (1.3%, n = 1337) exposed to a parental criminality, substance abuse and separation/single-parent household. PD was more common in classes 2–6 (Table 5). Individuals in these classes had a two–threefold risk of PD compared to class 1.

**Table 5:** Associations between latent classes of childhood adversity and PD

Latent classes	Total (n, column %)	PD	Model I^a^	Model II^b^	Model III^c^	Model IV^d^
Class 1: “No adversities”	58,750 (54.8)	291 (0.5)	1 (REF)	1 (REF)	1 (REF)	1 (REF)
Class 2: “Few with familial death, substance abuse and criminality”	29,076 (27.1)	232 (0.8)	1.6 (1.4–1.9)	1.5 (1.3–1.8)	1.4 (1.2–1.7)	1.3 (1.1–1.6)
Class 3: “Separation and public assistance”	11,855 (11.0)	135 (1.1)	2.3 (1.9–2.8)	2.2 (1.7–2.7)	1.8 (1.4–2.2)	1.4 (1.1–1.8)
Class 4: “Substance abuse, separation and public assistance”	5437 (5.1)	90 (1.7)	3.3 (2.6–4.2)	3.0 (2.3–3.9)	2.2 (1.7–2.9)	1.8 (1.4–2.4)
Class 5: “Substance abuse, and psychiatric morbidity”	832 (0.8)	9 (1.1)	2.2 (1.1–4.3)	1.9 (1.0–3.8)	1.6 (0.8–3.2)	1.6 (0.8–3.2)
Class 6: “Most adversities”	1337 (1.2)	13 (1.0)	1.9 (1.1–3.3)	1.8 (1.0–3.2)	1.6 (0.9–2.8)	1.4 (0.8–2.5)

Results from cox regression analyses, presented as hazard ratios (HR) with 95% confidence intervals (CI)

^a^Adjusted for birth year and sex

^b^Model I with additional adjustments for mother’s country of birth, parental income and education, and school performance

^c^Model II with additional adjustments for childhood/adolescent psychopathology [i.e. psychiatric diagnosis before age 18 years (PD excluded)]

## Discussion

### Key findings

To our knowledge, this is the first large-scale population-based study to examine the relationship between CA and the risk of being diagnosed with a personality disorder in young adulthood. Our study of more than 100,000 young adults showed evidence of the link between CA and diagnosed PD, a risk that increased in a dose–response manner as the number of CAs increased. Findings further suggest that the effect of CA on PD was partly modified by childhood psychopathology and school performance, such that individuals exposed to both had a multifold increased risk as compared to unexposed. Analyses further revealed that the association between CA and PD was partly mediated by school performance. Lastly, by means of LCA, we identified six subgroups of young adults with distinct patterns of exposure to CAs. We found that the class most closely related with diagnosed PD included individuals who had grown up with parental substance abuse, separation and public assistance.

The strengths of the present study include the large sample size, the longitudinal design, and high-quality register data on exposure, outcome and multiple potentially confounding factors. We use registers of high completeness and validity [39, 51]. The significant size of the cohort allowed for detailed analyses demonstrating the cumulative effect of CA and how it interacts with childhood psychopathology and school performance. Other studies with similar research questions have often been retrospective and based on self-reported information, entailing risk for recall bias (e.g., underreporting of CA) [52].

The use of population-based registers in studies of childhood adversities also comes with limitations. Relying solely on register data, there are adversities that we were not able to study. Probably the most decisive limitation with respect to CA is the lack of data on child maltreatment, includingabuse and neglect, adversities that have been pointed out as strong risk factors for PD.

In the studied cohort, 0.7% of participants received a diagnosis of PD during the follow-up period. This must be set in relation to the fact that few individuals with PD actually receive a formal diagnosis, as, in clinical practice, PD is seldom diagnosed [2], in spite of the fact that there is a point prevalence of 4–15% estimated in studies using self-reports or diagnostic interviews [1, 2, 53–56]. The register-based diagnoses capture cases of PD severe enough to receive a diagnosis made by a physician. Furthermore, a formal diagnosis of PD is often given only after a rather long clinical observation time. Since we had a short follow-up period in our study (the participants were only followed up until ages 20–24 years), our reported PD rate is most likely an underestimation of the true lifetime prevalence of PD in this cohort. This is supported by the data from our previous study based on Swedish national register data [6], where less than 30% of those given a diagnosis of PD in health care had obtained that diagnosis before the age of 25.

A consequence of the short follow up period is also that the association between CA and PD is only proven for those that have a PD diagnosis at an early age, here before the age of 25. As the type of PD is related to age, where antisocial, borderline, and passive-aggressive traits are seen in younger persons [57, 58], while schizoid features, schizotypal, avoidant, and obsessive–compulsive PDs and traits are more frequent in older age [55] our results cannot uncritically be generalized to those with a late debut PD.

Lastly, the current study is based on ICD-10 diagnoses, and although ICD has never included age restriction for diagnosis of PD, many physicians are concerned with diagnosing individuals at too young age, and ICD include instructions to be cautious with diagnosing too young people [2].

In this context it must be pointed out that the conceptual structure of the personality disorder group of conditions is currently under debate, an issue which has affected the development of both DSM-5 [59] and ICD-11 [2]. Both systems acknowledge the need for a dimensional system. In the forthcoming ICD-11 the subdivision into different domains is subordinated general criteria, and instead there is an attempt to dimensionalize the severity of the condition [2]. Nevertheless, a base requirement is fulfilment of the general criteria, which *in* sensu remain unchanged. In the present study all PDs have been analyzed together, i.e. with only the core general criteria in common.

Within the context of the above discussed limitations, our findings revealed that it is very common for children to grow up in adversity. Approximately half of the individuals were exposed to at least one CA, and 25% to two or more. These proportions are consistent with prior research in various settings, both from the US and Europe [22–24], showing that CA is a common phenomenon.

To date, most of existing studies examining the relationship between CA and PD have generally been based on small clinical samples [20, 21], and have used retrospectively self-reported adversities. With a large cohort, our findings revealed an association between a majority of the studied CAs and diagnosed PD. Earlier studies have shown a dose–response relationship between number of CAs and various types of psychiatric disorders [22–24, 60]. Our study extends previous research by demonstrating this association specifically for PD. Our findings show a strong link between increasing counts of adversities and PD in early adulthood, even after accounting for sociodemographic variables.

Prior research on sex differences in PD has been inconclusive. Some studies have concluded that women are at higher risk for PD, but other studies have shown the opposite [61]. There may be sex differences within the PD categories, with BPD more common in women, and antisocial PD more common in men [61, 62]. When stratifications were made (data not shown), PD rates were higher among women than men, findings similar to a recently published Danish study [62]. The effect of CA on PD was however similar for both sexes.

Our findings extend earlier work on CA and PD by also examining the role of childhood psycho pathology and school performance. Compared to the whole cohort, PD patients were more likely to have been treated with another psychiatric diagnosis prior to their 18th birthday. We found that a combination of CA and childhood psychopathology entailed highest risk of being diagnosed with PD in early adulthood. One explanation to this relationship could be that childhood psychopathology is an early reflection of PD. Experts has agreed that PD has its roots in childhood and adolescence [5, 30, 63], although it is highly unlikely to be diagnosed before age 16–17 years [5]. Of the 770 young adults diagnosed with PD in our cohort, only 16 individuals had a PD diagnosis before their 18th birthday. Thus, the early psychiatric diagnosis could reflect a PD onset.

Subsequently, it was more common for the PD patients to have lower school grades, and the analysis of CA, school performance and PD revealed that being exposed to CA and having lower school grades was particularly detrimental to PD. Analysis further revealed that school performance tend to mediate the association between CA and diagnosed PD. As CA increases the risk for PD, exposure to CA may set in motion mental health problems leading to worse school performance. Individuals with lower IQ tend to have more difficulties in coping with stressful life events, leading both to worse school performance [64] and higher risk for developing PD [31, 32].

Lastly, as an alternative approach to the cumulative risk measure, we used LCA to identify important subgroups with various patterns of coexisting CAs. These analyses revealed six classes, where the class most closely related with the risk for PD included individuals who had grown up with parental substance abuse, separation and public assistance.

## Conclusion

In conclusion, the present findings suggest that exposure to CA is strongly related to diagnosed PD in young adulthood. Individuals exposed to CA who fail in school or develop childhood and adolescent psychopathology are particularly at high risk for now being diagnosed with PD in young adulthood. Our findings indicate that special attention should be given in schools and health services to children exposed to these adversities to prevent decline in school performance, and to detect vulnerable individuals that may be on a negative life-course trajectory.

## Electronic supplementary material

Below is the link to the electronic supplementary material.

Supplementary Table 1 Model fit statistics for latent class analysis (DOCX 12 kb)

Supplementary Figure 1 Model of relationship between cumulative childhood adversity (CA), GPA, and personality disorder (PD). The value in parenthesis represents the coefficient for the direct (i.e. unmediated) path. (DOCX 164 kb)

Supplementary Figure 2 Conditional probabilities for latent classes. (PDF 16 kb)
