# Supplementary material for: Association between childhood adversity and a diagnosis of personality disorder in young adulthood: a cohort study of 107,287 individuals in Stockholm County
Source: Eur J Epidemiol. 2017 May 30;32(8):721–31. doi: 10.1007/s10654-017-0264-9 (PMC5591358; doi:10.1007/s10654-017-0264-9)
Supplement: Supplementary file 1 — Model fit statistics for latent class analysis (DOCX 12 kb) [file 10654_2017_264_MOESM1_ESM.docx]

| **Model** | **BIC(LL)** | **L²** | **Reduction in L²** | **Classification Error** |
| --- | --- | --- | --- | --- |
| 1 | 464,888 | 49,481 | 0.00% | 0,0000 |
| 2 | 420,882 | 5,382 | 89.12% | 0,0699 |
| 3 | 416,669 | 1,076 | 97.83% | 0,1519 |
| 4 | 416,409 | 723 | 98.54% | 0,1456 |
| 5 | 416,067 | 289 | 99.42% | 0,1771 |
| **6** | **416,051** | **180** | **99.64%** | **0,1929** |
| 7 | 416,086 | 123 | 99.75% | 0,3598 |
| 8 | 416,145 | 88 | 99.82% | 0,1790 |
| 9 | 416,243 | 94 | 99.81% | 0,1662 |
| 10 | 416,316 | 75 | 99.85% | 0,3690 |
