# Supplementary material for: Association between childhood adversity and a diagnosis of personality disorder in young adulthood: a cohort study of 107,287 individuals in Stockholm County
Source: Eur J Epidemiol. 2017 May 30;32(8):721–31. doi: 10.1007/s10654-017-0264-9 (PMC5591358; doi:10.1007/s10654-017-0264-9)
Supplement: Supplementary file 2 — Model of relationship between cumulative childhood adversity (CA), GPA, and personality disorder (PD). The value in parenthesis represents the coefficient for the direct (i.e. unmediated) path. (DOCX 164 kb) [file 10654_2017_264_MOESM2_ESM.docx]

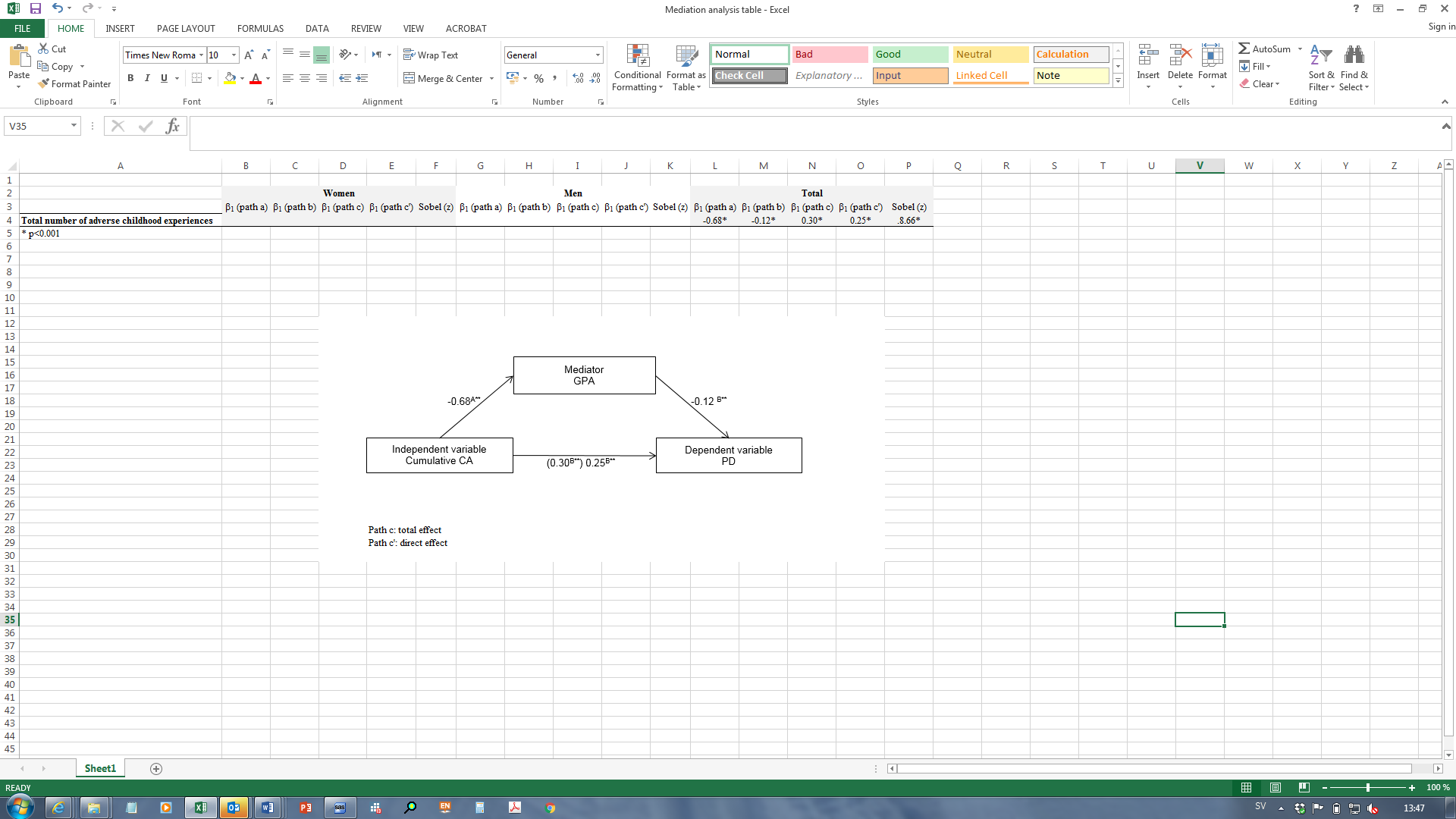


^A^ OLS regression coefficients: $\beta_{0}=14.69, \beta_{1}=-0.68CA$

^B^ Logistic regression coefficients: GPA 🡪 PD: $\beta_{0}=-3.33, \beta_{1}=-0.12\times GPA$

Total effect: Cumulative CA 🡪PD: $\beta_{0}=-5.03, \beta_{1}=0.30\times CA$

Direct effect: Cumulative CA 🡪PD: $\beta_{0}=-4.01, \beta_{1}=0.25\times CA$

** p<.0001
