# Supplementary figures and images for: Association between childhood adversity and a diagnosis of personality disorder in young adulthood: a cohort study of 107,287 individuals in Stockholm County
Source: Eur J Epidemiol. 2017 May 30;32(8):721–31. doi: 10.1007/s10654-017-0264-9 (PMC5591358; doi:10.1007/s10654-017-0264-9)

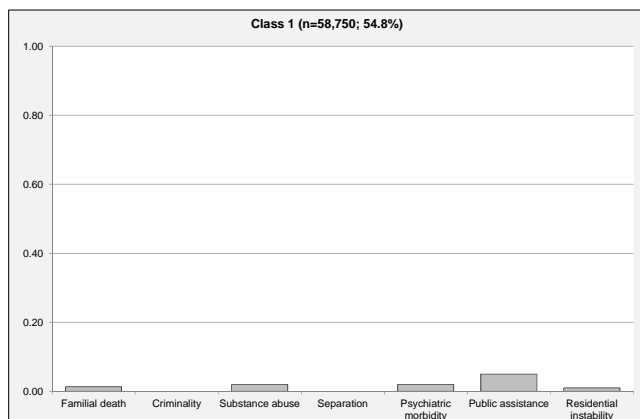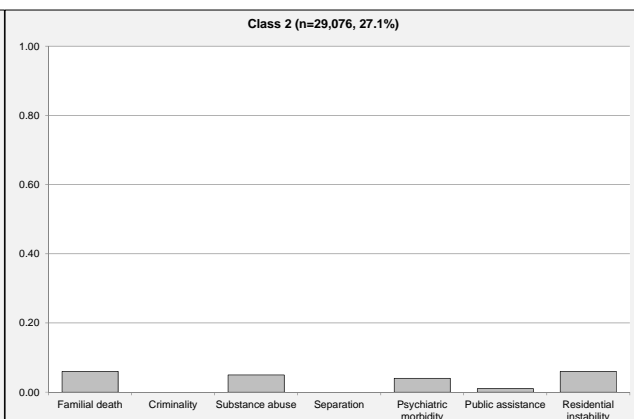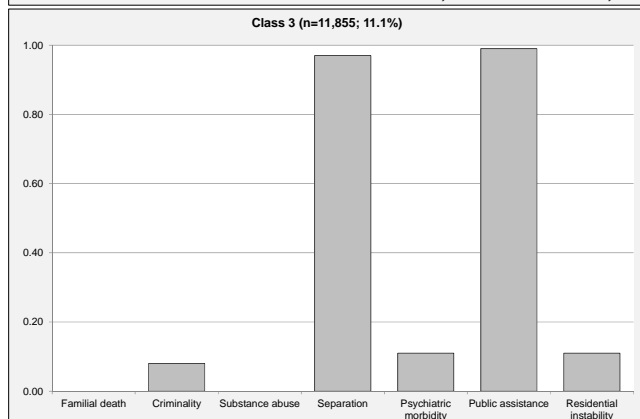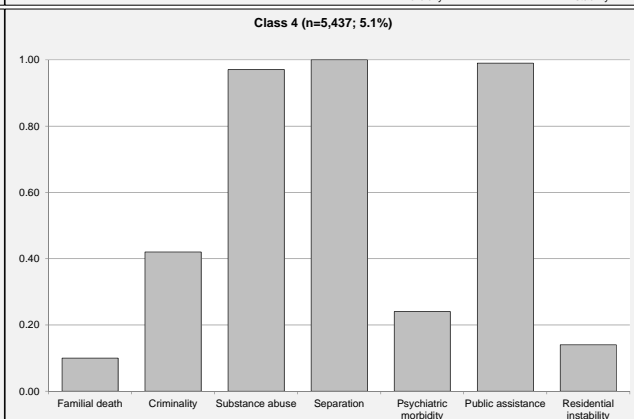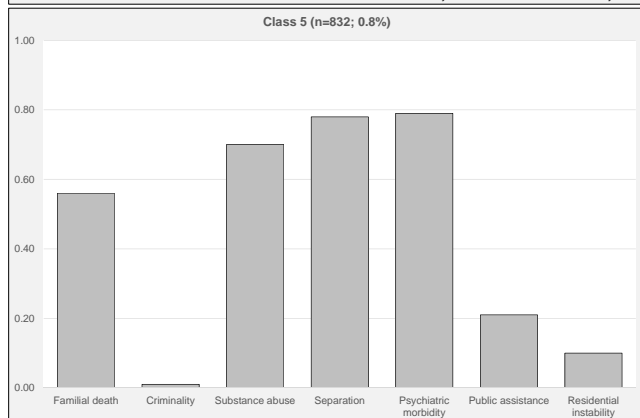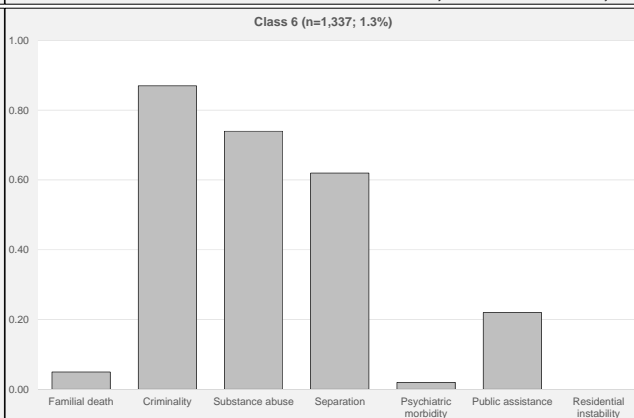

Supplement: Supplementary file 3 — Conditional probabilities for latent classes. (PDF 16 kb) [file 10654_2017_264_MOESM3_ESM.pdf]
